# Supplementary material for: A multi-method psychological autopsy study on youth suicides in the Netherlands in 2017: Feasibility, main outcomes, and recommendations
Source: PLoS One. 2020 Aug 27;15(8):e0238031. doi: 10.1371/journal.pone.0238031 (PMC7451645; doi:10.1371/journal.pone.0238031)
Supplement: S1 File — This is a 32-time checklist of the consolidated criteria for reporting qualitative studies (COREQ). (DOCX) [file pone.0238031.s001.docx]

**Consolidated criteria for reporting qualitative studies (COREQ): 32-item checklist**

Tong A, Sainsbury P, Craig J. Consolidated criteria for reporting qualitative research (COREQ): a 32-item checklist for interviews and focus groups. International journal for quality in health care. 2007 Dec 1;19(6):349-57.

**Domain 1: Research team and reflexivity**

Personal Characteristics

1. Interviewer/facilitator Which author/s conducted the interview or focus group?

Interviews conducted by authors Elias Balt, Milou Looijmans, Diana van Bergen, and three senior interviewers who are not authors but are credited in the acknowledgements: Henk van de Beld, Alice Schutte and Anne Roos.

1. Credentials What were the researcher’s credentials? E.g. PhD, MD

We mention authors’ credentials on the title page with authors’ affiliations.

1. Occupation What was their occupation at the time of the study

Saskia Mérelle: Senior researcher

Diana Van Bergen: Associate professor of Education and senior researcher

Milou Looijmans: Junior Researcher

Elias Balt: Junior Researcher

Sanne Rasing: Senior researcher

Lieke van Domburgh: Manager of a Residential Youth Care Unit

Maaike Nauta: Professor of Developmental Psychology

Onno Sijperda: Coroner

Wico Mulder: Youth doctor

Renske Gilissen: Manager at a Research Department

Gerdien Franx: General Manager

Daan Creemers: Clinical Psychologist and Research Coordinator

Arne Popma: Professor of Child and Adolescent Psychiatry and Child and Adolescent Psychiatrist

1. Gender Was the researcher male or female?

The research team and interview team consisted of slightly more females than males identified persons (60/40)

1. Experience and training What experience or training did the researcher have?

The researchers were trained as academics and/or mental health care professionals, and their background is in the behavioural sciences, social sciences and medical sciences.

Relationship with participants

1. Relationship established. Was a relationship established prior to study commencement?

In one case, parents who were part of the sample were also part of a series of test interviews. They were interviewed twice (the first time in the testing phase of the instrument, and the second time as part of the study, yet by different interviewers). In all other cases, the informants had never heard of the study nor had they met the research time prior to taking part in the study.

1. Participant knowledge of the interviewer

What did the participants know about the researcher? e.g. personal goals, reasons for doing the research.

The participants received an information letter that, amongst other aspects, explained who the research team and research institute were that conducted the study. The information letter was approved by the Medical Research Ethics Committee (MREC) of Amsterdam UMC.

1. Interviewer characteristics What characteristics were reported about the interviewer/facilitator? e.g. Bias, assumptions, reasons and interests in the research topic

We assume that the variety in gender, age, sexual orientation as well as the multidisciplinary research team and advisory board has helped us to overcome potential biases, as we held a series of reflection meetings during all phases of the research project.

**Domain 2: study design**

The answers to almost all questions for domain 2 and 3 (number 9 through 32) can be found on page 7 through 11 of the manuscript (Method section).

Theoretical framework

1. Methodological orientation and Theory

What methodological orientation was stated to underpin the study? e.g. grounded theory,

discourse analysis, ethnography, phenomenology, content analysis

See page 10 (Constant Comparative Method).

Participant selection

1. Sampling How were participants selected? e.g. purposive, convenience, consecutive, snowball

See page 7-8.

1. Method of approach How were participants approached? e.g. face-to-face, telephone, mail, email

See page 7-8.

1. Sample size How many participants were in the study?

See page 11-14.

1. Non-participation How many people refused to participate or dropped out? Reasons?

See page 11, 14-15.

Setting

1. Setting of data collection Where was the data collected? e.g. home, clinic, workplace

See page 8.

1. Presence of non-participants Was anyone else present besides the participants and researchers?

See page 8.

1. Description of sample What are the important characteristics of the sample? e.g. demographic data, date

See page 11-14.

Data collection

1. Interview guide. Were questions, prompts, guides provided by the authors? Was it pilot tested?

See page 9-10.

1. Repeat interviews. Were repeat interviews carried out? If yes, how many?

See page 8.

1. Audio/visual recording. Did the research use audio or visual recording to collect the data?

See page 8, 11.

1. Field notes Were field notes made during and/or after the interview or focus group?

See page 10.

1. Duration. What was the duration of the interviews or focus group?

See page 8.

1. Data saturation. Was data saturation discussed?

See page 9 and page 14.

1. Transcripts returned. Were transcripts returned to participants for comment and/or correction?

All informants were allowed to see the verbatim transcript of their interview, but not allowed to comment or correct the transcript.

**Domain 3: analysis and findings**

Data analysis

1. Number of data coders How many data coders coded the data?

See page 11.

1. Description of the coding tree Did authors provide a description of the coding tree?

The coding list is available in Dutch upon request of the first author.

1. Derivation of themes. Were themes identified in advance or derived from the data?

See page 11.

1. Software What software, if applicable, was used to manage the data?

See page 10.

1. Participant checking Did participants provide feedback on the findings?

Participants were offered the opportunity to read the research report in Dutch before publication. In two cases, participants provided feedback to the findings which were incorporated and adjusted for the present manuscript.

Reporting

1. Quotations presented. Were participant quotations presented to illustrate the themes / findings? Was each quotation identified? e.g. participant number

We choose not to include quotes in the present manuscript, for the purpose of keeping the amount of words at reasonable length. Since our manuscript offers a broad and comprehensive overview of all main patterns and themes in the entire dataset, we feel that adding quotations would result in a very lengthy paper, which arguably would be detrimental to the readability.

1. Data and findings consistent. Was there consistency between the data presented and the findings?

See result section on page 11-24.

1. Clarity of major themes. Were major themes clearly presented in the findings?

See page 11-24.

1. Clarity of minor themes. Is there a description of diverse cases or discussion of minor themes?

Our manuscript offers a broad and comprehensive overview of all main patterns and themes in the entire dataset. In the future, we will work on more in-depth presentations and elaborations of specific single themes that warrant further investigation.
