# Supplementary material for: A multi-method psychological autopsy study on youth suicides in the Netherlands in 2017: Feasibility, main outcomes, and recommendations
Source: PLoS One. 2020 Aug 27;15(8):e0238031. doi: 10.1371/journal.pone.0238031 (PMC7451645; doi:10.1371/journal.pone.0238031)
Supplement: S2 File — The English version of the interview guide that was used in the psychological autopsy study. (DOCX) [file pone.0238031.s002.docx]

INTERVIEW INSTRUMENT

Version: Parents

Dr. Diana van Bergen

Dr. Saskia Mérelle

Professor Arne Popma

***1: A few opening questions and the family background***

### **Interview**

1.First name of the deceased child:……………………………………………………………………

2. How is your family made up? (brothers, sisters, one or two biological parents, stepparents, etc.)

3. Where was he/she born? Where did he/she grow up?

*If born outside the Netherlands, how old was he/she when he/she came to the Netherlands*?

4. Can I assume that you are the biological parent, or is there a different relationship? (Ascertain whether the child was adopted)

5. Were you born in the Netherlands? If not, in which country?

6. Was your child born in the Netherlands? If not, in which country?

7. Did your child live with you at home? (Ask further questions to ascertain how/where the child lived)

8. If you (mother) work, may I ask what you do?

(If unemployed, note the period of unemployment and most recent job)

……………………………………………………………………………………………

9. If you (father) work, may I ask what you do?

(If unemployed, note the period of unemployment and most recent job)

**The death**

1. [Date of death (When did your child die?) ……../………/………
2. How were you informed?
3. [Method] How did your child die?

***2. Open narrative approach to perceived causes of the suicide***

[An open narrative approach in which the next-of-kin is able to recount in their own words all the factors that played a role in the development of the suicidality. The interviewer asks questions based on the next-of-kin’s account, but no new topics are broached]

1.Looking at the life of (*name of child*), can you tell me what factors you think played a role in their suicide?

[The following questions at the end, to learn from the suicide]:

2. Where the suicide came *OUT OF THE BLUE*: Looking back, do you think there was a particular moment when the problems might have been spotted?

3. Where the suicide was NOT OUT *OF THE BLUE*: And if we look back at how things unfolded, what do you wish could have happened differently?

For both *not out of the blue* and *out of the blue* suicide*:*

4. Were there things or problems that you wished had been tackled differently?

**3. Domain: Transition problems/Life stage problems**

**Background to the transition problems: Childhood development (birth to 11 years)**

*I’d like to ask you a few questions about his/her early childhood.*

1. How was his/her physical development during childhood?

2. How was he/she as a child?

You should cover at least the following topics:

- How were things at home?
- When he/she was little, what was his/her bond with his/her parents like? And with brothers/sisters?

(Further questions: Were there ever any problems between X (name) and you as their mother/father? Can you remember any such problems?)

- How did X do at school?

Further questions: Did he/she get on well at school? Was he/she happy at primary school?

(Further questions: If not, can you remember a time when X was unhappy? And: What made your child unhappy at that time?)

- How did he/she get on with other children?

(Further questions: Did he/she have friends? Was he/she bullied? Could you give an example of this? What happened with the bullying? How did it affect X? Was anything done about it?

1. Were things that X found difficult, or which made him/her sad or angry (*choose whichever applies*) discussed at home? Can you remember such a moment, perhaps give an example of this?
2. Some parents allow their children great freedom and have few rules; others are stricter and apply lots of rules. Could you tell me how that was in your home? (Further questions: Did you sometimes give X a reward? Did X sometimes receive punishment?)

**Adolescence – development (12 to 18 years)**

1.How was the start of your child’s puberty? AND afterwards?

(Further questions about changes that may have taken place: Earlier, you described how X (name of child) was when he/she was a child; did he/she change as he/she grew older and entered puberty?

2.How was your bond at that time (mother)? And how was your bond at that time (father)? What about the bond with brothers/sisters?

(Further questions: were there ever any problems between X (name) and you as X’s mother/you as X’s father/X’s brothers/sisters?

Further questions: did X talk to you about his/her feelings and thoughts? Were they feelings and thoughts that you would associate with suicidality?

3.How did X find the transition from primary school to secondary school?

4.How did X get on at secondary school?

Further questions: What were X‘s hobbies or interests? Thinking about the later suicide, did any of these ever strike you as odd or unusual?

Further questions: how did X’s schoolwork go as he/she grew older? Did you notice any change (in results, quality, etc.)? What seemed to be the reason for this change?

Further questions: did he/he/she have difficulty with schoolwork or need special help with learning (e.g. too much work or specific learning difficulties)?

Further questions: Did he/she eat properly?

Further questions: Did he/she sleep well?

5. How was X’s relationship with friends during puberty?

(Further questions: Did he/she have a friend whom he/she could trust/confide in? (for personal problems)?). Was he/she part of a group of friends, and what position did he/she have in it? Were there any changes or problems in the relationship with his/her friends?

6.Did he/she sometimes appear to be unhappy for extended periods during puberty?

7.If applicable: How did X find the transition from secondary school to further education?

8. [Autonomy/independence]: How independent did you feel he/she was? How dependent did you find him/her?

Further questions: Did he/she sometimes find it difficult to be a bit more independent? What were those difficulties? How was this apparent?

9. Did he/she go move out of the home to live alone? When? How did he/she find that? (Ask further questions about problems)

10. Do you know whether he/she ever went through a very traumatic experience?

(Ask in any event: Was he/she ever bullied? Was he/she badly affected by it? How did you notice that?

Did he/she ever suffer sexual abuse? Would you be able to say something about that? (Ask about burden, consequences and help)

11. Was there a sudden change in how happy he/she was whilst growing up?

(What was that sudden change; ask what this was related to)

(If not yet discussed: What is your fondest memory of X?)

**4. The last months (or month) before death**

{An open narrative approach in which the next-of-kin is able to recount in their own words what the victim’s life was like a few months prior to their death.}

1.Could you describe the last months before the death of your child? It would be helpful if you could mention everything that you think played a role in the run-up to their suicide.

2.Taking everything together, what do you think were the most important factors in those final months which led to his/her death?

*3.(Tipping points*) Was there a particular reason? Was there a straw that broke the camel’s back?

Further questions: were you aware at that time that this was a key moment in the life of your child?

**5. Suicidal Communication**

1. Was the suicide planned?

(Further questions: Had preparations been made? Did it seem sudden? (Probe with further questions)

2.Did he/she say anything which, with hindsight, suggests that he/she was planning suicide but the communication at the time was not that clear?

(For example, an attempt, or quietly saying goodbye, making certain statements or jokes). These could be things said to the parents, but also things the parents heard via others). It could also be expressed non-verbally, for example in drawings or an interest in certain music or films.

3. In the period before their death, did he/she speak to anyone about dying?

If yes: to whom, when and what was said?

What was their reaction?

4.Before their death, did he/she give a clear indication to someone that they were thinking of taking their own life?

5. Do you know how long ago your child first started thinking about suicide? (How old? Reasons?)

6. Did he/she leave a suicide note or an item behind for anyone?

(Further questions: Did the note explain the reasons for the death? For whom was the note/object intended?)

7. Do you know what mood he/she was in on the day of the death? How did this manifest itself?

1. **Domain: Social Media (incl. imitation, exposure, cyberbullying)**

Imitation

1.Did one of his/her friends ever try to harm themselves or take their own life? Was this someone from the offline group, or from an app group, etc.?

2. Did someone in your child’s network make positive statements about or express admiration for suicide? (Further questions: How did they do this? Was this an offline or online contact?)

3. Did the death/suicide of someone known to your child make a big impression on them?

4. Suicide or suicide attempts are sometimes reported in the media (TV, music, newspapers as well as online and on social media). Do you know whether he/she viewed any images or read any of these reports shortly before his/her death? (Further questions: How did you discover this? Did you speak about it to him/her)?

5. There was a great deal of attention in the Dutch media for the series *13 Reasons Why*; do you know this series, and did your son/daughter watch it? Did he/she talk to you about it? Did he/she also talk to you about the suicide scene in this series?

Social Media Use

6. Could you say something about your child’s use of social media? (ask further questions about Snapchat, Instagram. Facebook, but also *Netflix, games*;

(Further questions: Do you know which series your child watched and what games they played? Did you ever talk to your child about their content?)

7.Did anything strike you about your child’s profile photos? (Further questions: Had they chosen an unusual profile photo, or made changes to it?)

Social Media Online Contacts

8.Can you say something about your child’s online contacts?

(Further questions: Was your child a member of a particular group or community? Was this only an online group or did it also involve physical meetings?

Did your child have any negative experiences with social media? If so, what were they?

9.Do you know who your child followed online? Was your child following a person who had expressed suicidal ideation or who glorified suicide?

Social media bullying - Cyberbullying

10.Was your child ever subjected to social pressure online?

(Further questions: Could you say what form that took? Was your child challenged or persuaded online to do something that he/she didn’t want to do? Do you know whether he/she was ever abused by others online? Mention specifically: was this abuse sexual in nature?

If not yet addressed: Was social media use an important factor in your child’s suicide?

1. **DOMAIN LGBT and gender non-conformity**

**LGB**

1. Did your son or daughter ever tell you about someone that he/she fancied?

(Further questions: Do you happen to know whether he/she felt attracted to girls, boys or both? Did X ever fall in love? Could you say something about that? With a boy or girl? If a non-heterosexual preference: ask questions on the following themes.

Further questions:

- How did he/she feel after discovering this? Had he/she known about it for a long time?
- Did he/she come out? How did that go?
- Was he/she able to accept him/herself? Did he/she feel any shame?
- How did X feel about being LGB? Did X sometimes find it difficult to be LGB? Was X able to see a future for themselves as LGB?
- How did others react to his/her sexual orientation? What did you yourself think about it?
- Did he/she sometimes receive negative reactions?

1. Do you think your child’s sexual orientation was an important factor in his/her suicide? If so, in what way?

**Transgender**

1. Did your child’s birth gender correspond with how he/she felt (gender identity)?

Further questions:

- What was it like for him or her to discover this, and how did that go?
- Was he/she able to accept him/herself? Did he/she feel any shame?
- What did X feel about being transgender? Did X sometimes find it difficult to be a trans-boy/girl? Was X able to see a future for him/herself as a trans-boy/trans-girl?
- How did others react to his/her feelings (e.g. family, friends, etc.)? How did you yourself react?
- Did he/she sometimes receive less positive reactions from others?
- Did a medical or social transition take place? How did that go?

(N.B.: Pick up the questions about help here if applicable)

1. Do you think your child’s feelings (about being transgender) were an important factor in them taking their own life? If so, in what way?

**Gender non-conformity**

1. How ‘boyish’ (masculine) or ‘girlish’ (feminine) did you feel your son/daughter was? Further questions:

- How did he/she feel about not being completely ‘boyish’/’girlish’? Was he/she able to accept him/herself? Did he/she feel any shame or embarrassment?
- How did others react to his/her lack of masculinity/femininity? What did you feel about it?
- Did he/she sometimes receive less positive reactions from others?

1. Might his femininity (her masculinity) have been a factor in them taking their own life? If so, in what way?

**8.Domain: Medical and psychiatric history, and help**

1. Was help sought from counsellors or care professionals? If not: was help offered by counsellors or care professionals? What were the reasons for this? (Also check if suicidality was a reason). If not, ask why not, and explore the role played by culture and shame.

If help was sought/offered, continue to item 2.

1. Was your child receiving treatment from a care professional at the time of their death?

OR did your child receive counselling or support?

What kind of treatment and what kind of care professional? Or: What kind of counselling?

Was the help provided in an enforced or voluntary setting?

1. What was the focus of the treatment?

(Further questions: Did the care professional make a diagnosis?

Was medication prescribed and taken? Which medication?

Did your child also take non-prescribed medication?

1. Could you tell me how the treatment went? (What did he/she feel about the help?)

Further questions:

- Was the help appropriate for the situation? Was the help offered a good match for what your child needed? (Also compare the explanatory models for the illness of parents versus care professionals; check whether this might be due to cultural differences)
- Was help provided in time (waiting lists)?
- Was more than one professional involved? What was the cooperation like, and the handovers between different professionals?
- Was there a good connection with the professional? Not just between the professional and your child, but also between the parents and the professional?
- How often did your child have a session with the professional? Weekly? Monthly?
- Did your child receive sufficient help? Was he/she heard, seen, listened to by the care professional?
- Were you involved as parents in the treatment? If so, in what way? Was your child content with this? And you yourselves?
- Did the treatment produce any results? What were they? Was your child content with this? And you yourselves?
- How did the treatment conclude?

1. Were there things that could have gone better with this treatment? If so, what were they?

Concluding question: If not yet covered: Did the professional help play a role in your child’s suicide?

General: the questions above are intended as a guide, but it is key that the interviewer is fully aware of the treatment guidelines and tries to obtain an understanding of the quality and continuity of care. Also ask about after-care, for example: was there a relapse prevention plan in place after treatment (e.g. after the first treatment for depression/anxiety)?

**9.Domain: Non-Dutch / Migration background**

**Ethnic and Cultural identity**

1.Did your child seek contacts (e.g. friends) within his/her own culture, or outside their own culture?

2.Was he/she interested in the ____culture?

3. Did he/she feel Dutch, _____(fill in ethnicity), or both?

4.How did he/she feel about growing up in the Netherlands with a (fill in culture) background?

(Further questions: Did he/she sometimes find it complex growing up in a mix of different cultures?)

5. Was religion important for your child? (Further questions: Did your child go (or accompany you) to (place of worship)?

(Further questions: Was religion ever a source of argument between you and your child?)

6. Did you ever ask for help for your child from a spiritual leader (pandit, etc..)? If so, what help did you seek? (Further questions: how did this go?)

**Discrimination and feeling at home**

1.Did he/she feel at home in (town/city) the Netherlands? Did he/she feel at ease?

2.Did he/she sometimes feel that he/she was not treated fairly in the Netherlands because of his/her origin, skin colour or religion? What happened? Did this affect him/her?

3.Do you think these feelings were an important factor in the suicide?

**Acculturation and acculturation differences between parents and child**

1.Did you feel that your child did not sufficiently identify with their (____ethnicity)/behave as required in the____culture?

(Further questions: Did this ever give rise to conflict at home?

2.Did you feel that your child had become too ‘Dutch’? (Further questions: did this ever give rise to conflict at home?)

3. Do you think these things were an important factor in the suicide?

**Honour-related issues**

1.Did your son/daughter ever do anything which you felt was inappropriate in your culture or religion?

2. Did your son or daughter ever do anything that was a scandal?

3. Did your son or daughter ever display behaviour with which you had difficulty, in view of your culture or religion? (Further questions: Did your son or daughter ever behave in a way that was too free in your opinion?)

4. Do you think these things were an important factor in the suicide?
