# Supplementary material for: A multi-method psychological autopsy study on youth suicides in the Netherlands in 2017: Feasibility, main outcomes, and recommendations
Source: PLoS One. 2020 Aug 27;15(8):e0238031. doi: 10.1371/journal.pone.0238031 (PMC7451645; doi:10.1371/journal.pone.0238031)
Supplement: S3 File — The Dutch version of the interview guide that was used in the psychological autopsy study. (DOCX) [file pone.0238031.s003.docx]

INTERVIEW INSTRUMENT

Versie: Ouders

Dr. Diana van Bergen

Dr. Saskia Mérelle

Professor Arne Popma

***1: enkele startvragen en de familie achtergrond***

### **Interview**

1.Voornaam van de het overleden kind:……………………………………………………………………

2. Hoe ziet u gezin er uit? (broers, zussen, een of twee biologische ouders, stiefouders etc)

3. Waar is hij/zij geboren? Waar opgegroeid?

*Indien geboren buiten Nederland*, hoe oud was hij/zij toen hij/zij naar Nederland kwam?

4. Mag ik aannemen dat u de biologische ouder bent of ligt dat anders ? (Uitzoeken of er sprake is van adoptie)

5. Ben u in Nederland is geboren? Zo nee, uit welk land?

6. Is uw kind in Nederland geboren? Zo nee, in welk land?

7. Woonde uw kind thuis (doorvragen hoe het kind woonde)

8. Heeft u (moeder) werk, mag ik vragen wat u doet?

(Indien werkloos, noteer de periode van werkloosheid en de laatste baan.)

……………………………………………………………………………………………

9. Heeft u (vader) werk, mag ik vragen wat u doet?

(Indien werkloos, noteer de periode van werkloosheid en de laatste baan)

**Het overlijden**

1. [Datum van overlijden]Wanneer is uw kind overleden? ……../………/………
2. Hoe bent u op de hoogte gebracht?
3. [Methode] hoe is uw kind overleden?

***2. Open narratieve benadering van gepercipieerde oorzaken van de suïcide***

[Een open narratieve benadering waarin de nabestaande in eigen woorden kan vertellen wat er allemaal een rol heeft gespeeld bij het ontstaan van de suïcidaliteit. Er wordt doorgevraagd op het aangeboden verhaal, maar er worden nog geen nieuwe topics uitgevraagd]

1.Kijkend naar de levensloop van (*naam kind*), kunt u mij vertellen wat u denkt dat er allemaal een rol heeft gespeeld bij de zelfdoding ?

[Aan het einde de volgende vragen om te leren van de suïcide: ]

2. BIJ *OUT OF THE BLUE*: Als we terug kijken, was er dan volgens u een moment dat de problemen opgemerkt hadden kunnen worden

3. BIJ NIET *OUT OF THE BLUE*: En als we dat beloop zo bekijken, wat zou u wensen dat er toen anders was gelopen?

Bij zowel *niet out of the blue* als *out of the blue:*

4. Zijn er dingen of problemen waarvan u gewild had dat deze anders waren aangepakt?

**3. Domein Transitieproblematiek / Levensfase problematiek**

**Aanloop naar de transitie problemen: Kinderlijke ontwikkeling (geboorte tot 11 jaar)**

*Ik zou nu graag enkele vragen stellen over zijn/haar vroege kindertijd.*

1. Hoe ging het met de lichamelijke ontwikkeling in de kindertijd?

2. Hoe was hij/zij als kind?

In ieder geval ook stil staan bij de volgend onderwerpen:

- Hoe was het bij jullie thuis?
- Toen hij /zij klein was, hoe was toen de band met ouder(s)? En met broers/zussen?

(doorvragen: Was er ooit wel eens een probleem tussen X (naam) en u als moeder / u als vader? Kunt u zich nog zo’n probleem herinneren?)

- Hoe ging het bij X op school?

Doorvragen: Kond hij/zij goed meekomen op school? Was hij/zij gelukkig op de lagere school?

(doorvragen Indien neen, kun u zich nog een ongelukkig moment van X herinneren? E : wat maakte uw kind toen ongelukkig?)

- Hoe ging de omgang met andere kinderen?

(Doorvragen: Had hij/zij vrienden? Werd hij /zij gepest? Kunt u daar een voorbeeld van geven, hoe verliep dat pesten? Hoe was X daaronder? Werd er iets tegen gedaan?

1. Werden dingen die X moeilijk, verdrietig of wat hem/haar boos maakte (*kiezen wat van toepassing was*) thuis besproken? Kunt u zich een moment herinneren, daar een voorbeeld van geven?
2. Sommige ouders vinden veel goed en hebben weinig regels, anders ouders zijn wat strenger en hebben meer regels. Kunt u vertellen hoe u dat bij u thuis ging? (doorvragen: gaf u X wel eens beloning? Kreeg X wel eens straf?)

**Adolescentie – ontwikkeling (12 tot 18 jaar)**

1.Hoe verliep de start van de puberteit van uw kind? EN daarna?

(doorvragen naar wisselingen en veranderingen die mogelijk hebben plaatsgevonden: U heeft hiervoor beschreven hoe X (naam kind) was toen hij/ zij kind was, veranderde hij/zij toen hij/zij ouder werd en in de puberteit kwam?

2.Hoe was uw (moeder) band toen? En hoe was uw (vader) band toen? En met de broers /zussen?

(Doorvragen: Was er ooit wel eens een probleem tussen X (naam) en u als moeder / u als vader/ de broers/zussen

Doorvragen: Sprak X met u over zijn/haar gevoelens en gedachten? Waren die dit ook gevoelens en gedachten die u in verband brengt met suïcidaliteit?)

3.Hoe vond X het om van de basisschool naar de middelbare school te gaan?

4.Hoe ging het met X op de middelbare school?

Doorvragen: Wat waren hobbies of interesses? Hebt daar ooit iets opvallends aangezien, denkend aan de latere suïcidaliteit?

Doorvragen: Hoe ging het met zijn/haar schoolwerk toen hij/zij ouder werd? Was daar een verandering in te zien (resultaten, kwaliteit etc)? Wat leek de reden te zijn voor deze verandering? )

Doorvragen: Had hij/zij moeite om het schoolwerk uit te voeren of had hij/zij speciale hulp nodig bij het leren (vb. teveel werk of specifieke leerproblemen)?

Doorvragen: At hij/zij goed?

Doorvragen: Sliep hij/zij goed?

5. Hoe was de band met vrienden in de puberteit?

(Doorvragen: Had hij/zij een vriend(in) die hij/zij kon (toe)vertrouwen? (voor persoonlijke problemen) ?) Maakte hij/zij deel uit van een vriendengroep en welke positie had hij/zij daarin? Waren er ooit veranderingen of problemen in de relatie met zijn vrienden

6.Leek hij/zij soms ongelukkig voor een langere periode in de puberteit?

7.Indien van toepassing : Hoe vond X het om van de middelbare school naar zijn/haar vervolgopleiding te gaan?

8. [Autonomie/zelfstandigheid]: Hoe zelfstandig vond u hem/ haar? Hoe afhankelijk vond u hem/haar?

Doorvragen Heeft hij /zij het wel eens moeilijk gevonden om wat zelfstandiger te worden? ? Welke, hoe bleek dit?

9. Is hij / zij op zich zelf gaan wonen? Wanneer? Hoe vond hij/zij dat ? (doorvragen naar problemen)

10. Weet u of hij/zij ooit een erg ingrijpende gebeurtenis meegemaakt?

(In ieder geval uitvragen: is hij/zij ooit gepest? Had hij/zij er veel last van? Hoe heeft u dat gemerkt?

Is hij/zij eens seksueel misbruikt? Wilt u daar iets over vertellen (vragen naar last, gevolgen, en hulp)

11. Is er een knik geweest in hoe gelukkig zij/hij was tijdens het opgroeien?

(Hoe zag die knik eruit, doorvragen, waar had dit mee te maken)

(Indien nog niet besproken: Wat is uw mooiste herinnering aan X?)

**4. De laatste maanden (of maand) voorafgaand voor het overlijden**

{Een open narratieve benadering waarin de nabestaande in eigen woorden kan vertellen hoe het leven van het slachtoffer eruit heeft gezien enkele maanden voorafgaand aan het overlijden.}

1.Kunt u de laatste maanden beschrijven voorafgaand aan het overlijden van uw kind, en hierbij alles noemen wat volgens u een rol heeft gespeeld in de aanloop naar de suïcide?

2.Alles bij elkaar genomen, wat denkt u dat in deze laatste maanden de belangrijkste redenen zijn die geleid hebben tot zijn/haar overlijden?

*3.(Tipping points*) Was er een aanleiding ? Was er een druppel die de emmer deed overlopen?

Doorvragen; Was u zich op dat moment er van bewust dat dit een kernmoment was in het leven van uw kind?

**5. Suïcidale Communicatie**

1. Was de suïcide gepland ?

(doorvragen: waren er voorbereidingen getroffen? Leek het plotseling? (uitvragen)

2.Heeft hij/zij iets gezegd dat, achteraf gezien, de indruk geeft dat hij/zij de zelfdoding aan het plannen was maar dat op het moment van de communicatie niet zo duidelijk was?

(Bijvoorbeeld zoals een poging of stilletjes afscheid nemen bepaalde uitspraken of grappen maken). Het kan gaan om uitingen richting de ouders, maar ook wat ouders via via gehoord hebben). Kan ook niet verbaal tot uiting komen in tekeningen of belangstelling voor bepaalde muziek of films

3. Heeft hij/zij met iemand over sterven gesproken voor het overlijden?

Indien ja, tegen wie, wanneer en wat is er gezegd geweest?

Wat was hun reactie?

4.Heeft hij/zij voor het overlijden tegen iemand duidelijk aangegeven dat hij/zij eraan dacht om zichzelf te doden?

5. Weet u hoelang geleden uw kind voor het eerst begon te denken aan zelfdoding? (hoe oud? Aanleiding?

6. Liet hij/zij een afscheidsbrief/object na voor iemand?

(doorvragen: Werden er redenen voor het overlijden gegeven in de brief? Voor wie was de brief/object bestemd?

7. Weet u in welke stemming hij/zij was op de dag van het overlijden? Hoe uitte dit zich?

1. **Domein: Social Media (incl. imitatie, exposure, cyberbullying)**

Imitatie

1.Heeft één van zijn/haar vrienden ooit geprobeerd om zichzelf te beschadigen of suïcide te plegen? Betrof het iemand uit de offline of juist *app* groep etc?

2. Zijn er positieve woorden of bewondering uitgesproken over zelfdoding door iemand in de omgeving van uw kind? (Doorvragen: Op welke manier? was het een offline of online contact? )

3. Heeft het overlijden/de zelfdoding van een bekend persoon veel indruk gemaakt op uw kind?

4. Zelfdoding of suïcidepogingen worden wel eens weergegeven in de media (zowel tv, muziek, kranten maar ook online en social media), weet u of hij/zij hier beelden van heeft bekeken kort voor zijn/haar overlijden?(doorvragen hoe bent u daar achter gekomen, heeft u erover gesproken met hem/haar)?

5. In de media is veel aandacht geweest voor de serie *13 Reasons why*; kent u deze serie en heeft uw zoon/dochter deze bekeken? Heeft hij/zij hierover gesproken? Heeft hij /zij ook met u over de zelfdoding scene in deze serie gesproken?

Social Media Gebruik

6. Kunt u iets vertellen over het sociale media gebruik van uw kind? (doorvragen op Snapchat, Instagram. Facebook, maar ook *netflix, games*;

(doorvragen weet u welke series en games uw kind bekeek?] Sprak u wel eens met uw kind over de inhoud ervan?)

7.Was er u iets opgevallen aan de profiel foto’s van uw kind (Doorvragen: Was er een opvallende keuze voor profielfoto, of wijzigingen daarin?)

Social Media Online Contacten

8.Hoe verliepen de online contacten?

(doorvragen: Maakte uw kind deel uit van een bepaalde groep of community? Was dit alleen een online groep of ook met echte ontmoetingen?

Heeft uw kind negatieve ervaringen gehad met sociale media? Zo ja, welke?

9.Weet u wie uw kind online volgde? Was u kind een volger van een persoon die zich suïcidaal uitte of zelfdoding verheerlijkt?

Social media pesten - Cyber bullying

10.Is er ooit sprake geweest van sociale druk op uw kind online

(Doorvragen: Kunt u vertellen op welke manier? Is uw kind online uitgedaagd of overgehaald tot iets dat hij/zij niet wilde? Weet u of hij/zij ooit gebruikt werd door anderen online? Specifiek noemen: was dit seksueel van aard?

Indien nog niet aan bod: Was sociale media gebruik van belang voor de zelfdoding van uw kind?

1. **DOMEIN lhbt en gender non-conformiteit**

**LHB**

1. Heeft u zoon of dochter wel eens verteld over iemand die hij/zij leuk vond?

(doorvragen: Weet u misschien of hij/zich aangetrokken voelde tot meisjes, jongens of allebei?: Was X wel eens verliefd? Kun je daar iets over vertellen? Op een jongen of een meisje). Indien een niet-heteroseksuele voorkeur: doorvragen met onderstaande thema’s.

Doorvragen:

- hoe was het voor hem of haar om daar achter te komen? Liep hij/zij daar lang mee rond?
- heeft hij /zij een *coming out* gedaan? Hoe ging dat?
- kon hij/zij zichzelf accepteren, was er sprake van schaamte?
- Hoe vond X het om lhb te zijn? Vond X het wel eens moeilijk om lhb te zijn? kon X een toekomst voor zichzelf zien als lhb?
- Hoe reageerden andere op zijn/haar geaardheid? Hoe vond u het zelf?
- Kreeg hij/zij wel eens negatieve reacties?

1. Denk u dat de geaardheid van uw kind belang was voor de zelfdoding van uw kind? Zo ja hoe?

**Transgender**

1. Kwam het geboortegeslacht bij uw zoon/ dochter overeen met hoe hij/zij zich voelde (identiteit)?

Doorvragen:

- hoe was het voor hem of haar om daar achter te komen hoe ging dat?
- kon hij/zij zichzelf accepteren, was er sprake van schaamte?
- Hoe vond X het om trans te zijn? Vond X het wel eens moeilijk om transjongen/meisje te zijn? kon X een toekomst voor zichzelf zien als transjongen/transmeisje?
- Hoe reageerden anderen op zijn/haar gevoelens? (zoals familie, vrienden etc) Hoe vond u het zelf?
- Kreeg hij/zij wel eens minder leuke reacties van anderen?
- Is er sprake geweest van een medische of sociale transitie? Hoe is dat verlopen?

(let op, hier eventueel de hulpverleningsvragen oppakken)

1. Denk u dat de gevoelens (transgender) van belang waren voor de zelfdoding? Zo ja hoe?

**Gender non conformiteit**

1. Hoe jongensachtig (of hoe meisjesachtig) vond u uw zoon (dochter)? Doorvragen:

- hoe was het voor hem of haar om niet helemaal (weinig) jongensachtig/ meisjes achtig te zijn? kon hij/zij zichzelf accepteren? was er sprake van schaamte?
- Hoe reageerden anderen op zijn (weinig) jongensachtigheid/meisjesachtigheid? Wat vond u ervan?
- Kreeg hij/zij wel eens minder leuke reacties van anderen?

1. Zou zijn meisjesachtigheid (haar jongensachtigheid) van belang voor de zelfdoding? Zo ja, hoe?

**8.Domein Medisch en psychiatrisch verleden, en hulpverlening**

1. Is er hulp gezocht bij begeleiders of hulpverleners? Indien nee: is er hulp aangeboden door begeleiders of hulpverleners? Wat was de aanleiding? (check ook of suïcidaliteit reden was)

Indien nee, uitvragen waarom niet, en hierbij de rol van cultuur en schaamte verkennen.

Indien er wel sprak was hulp, door met 2.

1. Was uw kind onder behandeling van een hulpverlener op het moment van overlijden?

OF kreeg uw kind begeleiding of ondersteuning?

Wat voor soort behandeling en hulpverlener? Of : Wat voor soort begeleiding?

Was er sprake van hulp in een gedwongen of vrijwillige variant?

1. Waar was de behandeling op gericht?

(doorvragen: Is er een diagnose gesteld door de hulpverlener?

Werd er voorgeschreven en medicatie genomen? Welke?

Werd er ook niet voorgeschreven medicatie genomen?

1. Kunt u mij vertellen hoe de behandeling is verlopen? (Hoe stond hij/zij tegenover de hulp?

Door vragen:

- Was het passende hulp? Sloot de geboden hulp goed aan bij wat uw kind nodig had? (vergelijk ook de verklaringsmodellen voor de ziekte van ouders versus de hulpverleners, check of dit eventueel door culturele verschillen komt)
- Kwam er op tijd hulp (wachtlijsten)
- Was er sprake van meerdere behandelaars? Hoe verliep de samenwerking, en de overdracht tussen verschillende behandelaars?
- Was er een goede klik met de behandelaar? Tussen behandelaar en uw kind, maar ook tussen ouders en de behandelaar?
- Hoe vaak had uw kind een sessie met de behandelaar? Was dit wekelijks? Maandelijks?
- Werd u kind voldoende geholpen, gehoord, gezien, naar hem/haar geluisterd door de hulpverlening?
- Werd u als ouders betrokken bij de behandeling, zo ja hoe? Was uw kind u daar tevreden mee? En u zelf?
- Waren er resultaten van de behandeling? Welke? Was uw kind daar tevreden mee? En u zelf?
- Hoe verliep de afsluiting van de behandeling?

1. Waren er zaken die beter hadden gekund bij deze behandeling? Zo ja, wat?

Slotvraag: Indien nog niet aan bod: Speelde de hulpverlening een rol bij de zelfdoding van uw kind?

Algemeen: de vragen hierboven zijn richtinggevend maar de interviewer moet vooral goed op de hoogte zijn van de behandelrichtlijnen en proberen zicht te krijgen op kwaliteit, continuïteit van zorg. Ook nazorg navragen bijvoorbeeld; was er na uitschrijven een terugvalpreventieplan (eg na de eerste behandeling voor depressie/angst).

**9.Domein: Niet-Nederlandse/ Migranten Jongeren**

**Etnische en Culturele Identiteit**

1.Zocht uw kind contacten (bijv. vrienden) binnen eigen cultuur of juist buiten eigen cultuur?

2.Had hij /zij interesse in de ____cultuur?

3. Voelde hij/zij zich Nederlands, _____(etniciteit invullen), of allebei?

4.Hoe vond hij/zij het om met een (cultuur invullen) afkomst op te groeien in Nederland?

(doorvragen: Vond hij/zij het wel eens ingewikkeld om in verschillende culturen op te groeien?)

5. Was het geloof belangrijk voor uw kind? (doorvragen: Ging uw kind (mee) naar het (gebedshuis)?

(doorvragen: Was het geloof wel eens reden voor ruzie tussen u en uw kind?)

6. Heeft u weleens hulp gevraagd voor uw kind aan een geestelijk leider (pandit enz.)? Zo ja, wat was de vraag? (doorvragen: hoe is dit verlopen?)

**Discriminatie en thuisvoelen**

1.Voelde hij / zij zich thuis in (stad) Nederland? Voelde hij/zij zich op zijn/haar gemak ?

2.Vond hij/zij wel eens dat hij/zij niet eerlijk werd behandeld in Nederland vanwege afkomst, huidkleur of geloof? Wat gebeurde er? Had hij/zij daar last van?

3.Denkt u dat deze gevoelens belangrijk waren voor de zelfdoding?

**Acculturatie en acculturatie verschillen ouders en kind**

1.Vond u dat uw kind zich nog genoeg (____etniciteit) voelde/ gedroeg zoals nodig in de____cultuur?

(Doorvragen: Is daar wel eens een conflict over geweest thuis?

2.Vond u dat uw kind te Nederlands is geworden? (doorvragen: Is daar wel eens een conflict over geweest thuis?)

3. Denkt u dat deze dingen belangrijk waren voor de zelfdoding?

**Eergerelateerde problematiek**

1.Heeft uw zoon/dochter wel eens iets gedaan dat u niet vond passen bij uw cultuur of geloof?

2. Heeft uw zoon of dochter wel eens iets gedaan dat een schande was?

3. Heeft uw zoon of dochter wel een gedrag laten zien waar uw moeite mee had, gelet op uw cultuur of geloof? (doorvragen: Heeft uw zoon of dochter zich wel eens te vrij gedragen naar uw mening?

4. Denkt u dat deze dingen belangrijk waren voor de zelfdoding?
